# Assessment of Disability and Factors Determining Disability among Inhabitants of South-Eastern Poland Aged 71–80 Years

**DOI:** 10.1155/2018/3872753

**Published:** 2018-06-19

**Authors:** Agnieszka Ćwirlej-Sozańska, Bernard Sozański, Agnieszka Wiśniowska-Szurlej, Anna Wilmowska-Pietruszyńska, Jolanta Kujawa

**Affiliations:** ^1^Department of Medicine, Institute of Physiotherapy, University of Rzeszow, Rzeszow, Poland; ^2^Center for Innovative Research in Medical and Natural Sciences, University of Rzeszow, Rzeszow, Poland; ^3^Department of Medical Rehabilitation, Military Medical Faculty, Medical University of Lodz, Lodz, Poland

## Abstract

**Introduction:**

The aim of the study was to assess the level of disability in a sample of older people in south-eastern Poland and to identify any potential relationship between their profile of functioning and a complex set of variables including activities, participation, and contextual factors.

**Materials and Methods:**

The study included 800 people aged 71–80 years. The WHODAS 2.0 questionnaire was applied for the assessment of disability.

**Results:**

A total of 43.88% of the interviewees showed a moderate level of disability, while 17.75% had severe or extremely high levels of disability. In addition, 7.75% of the interviewees had no functional limitations and 30.62% demonstrated a mild level of disability. The studied individuals reported the greatest difficulties with life activities such as cleaning, cooking, or shopping, followed by Limited Participation and then getting along. Age, number of chronic diseases, a low level of education, a low level of physical activity, poor living conditions, and lack of opportunities for daily help significantly contributed to higher levels of disability.

**Conclusions:**

Measures intended to reduce the level of disability in older adults should focus on improving medical care, health education, increasing physical activity, adapting housing to the needs of everyday functioning, and providing daily help.

## 1. Introduction

Aging is a progressive and irreversible process for all living organisms [1]. In Europe, the percentage of residents aged 65 years and over will have increased from 18.9% to 29.5% [2] by 2016, and the proportion of those aged at least 80 years from 4.6% to 12% [3]. Polish Central Statistical Office (CSO) figures indicate that the percentage of the population of Poland aged 65 years and over (13.5% in 2014) will have increased to 34.5% by 2050 [4].

As the efficiency of physiological mechanisms declines with age, human functional efficiency decreases, and the number of health problems increases [5], resulting in a greater occurrence of functional limitations and multimorbidity in older than younger people [6]. The aging of the population is accompanied by an increase in the number of disabled people and dependents [7]. In an analysis of three large longitudinal surveys, Chatterji et al. [8] suggest that older people in most European countries experience difficulties in performing basic and instrumental activities of daily living. They also suggest that while the performance of the basic activities of daily living is gradually improving, there is no clear evidence that the limitations associated with the instrumental activities of daily living are being removed. The PolSenior study found people aged over 70 years to have increasing problems with performing complex everyday activities [9]. According to Salomon et al. [10], average life expectancy increased between 1990 and 2010 in 187 countries, but the number of years lived in good health increased to a lesser degree. The authors found that, during the 20-year period before the study, no progress was made in reducing the impact of chronic disease on the health and functional status of the population.

In response to the need for a functional description of disability, the WHO developed the International Classification of Functioning, Disability and Health (ICF) [11]: a biopsychosocial model of disability encompassing medical issues, activity and participation in community life, as well as environmental and personal factors. ICF-based tools allow the severity of problems to be evaluated in both the individual and the entire population [12].

Based on the ICF, the WHO developed Disability Assessment Schedule-3.6 (WHODAS 2.0): a high-quality disability assessment tool for epidemiological and clinical studies. WHODAS 2.0 shows very good psychometric properties [13] and has been confirmed as an instrument for measuring disability in many countries, including Taiwan [14], Spain [15], Turkey [16], Iran [17], China, Russia, and India [18]. The tool allows areas of personal and social functioning that require support to be identified [19]. Frederici et al. found the questionnaire to be suitable for evaluating the state of health and disability in a range of populations [20]. Disability assessment in the place of residence is valuable as it allows the problems associated with everyday life functioning to be highlighted, and the findings are of value for the social services, care, and health services [12].

The prevalence of disability has been found to vary according to country. A study based on WHODAS by Olaya et al. found that level of disability in those aged over 50 years to be the highest in Poland, followed by Spain and Finland [21]. Further studies performed on seniors living in rural areas in Poland found 28.26% of the study group to have at least a moderate level of disability. The authors also note the presence of relationships between disability and age, sex, level of education, and physical activity [22]. In addition, Pareles et al. report that education, marital status, and profession have the strongest relationships with active aging [23].

To address the need to unify the disability evaluation process, studies using tools based on the ICF have been performed to measure individual functioning and disability, among seniors aged between 71 and 80 years. An increase in the proportion of the population aged over 70 years has been observed in Poland [24], and this group is particularly exposed to the growth in the prevalence of disability [25]. The aim of the present study was to evaluate the level of disability and identify any potential relationship between level of disability and a set of variables, including basic sociodemographic characteristics, number of chronic diseases, BMI, physical activity, social engagement, and living conditions, in older people in south-eastern Poland.

## 2. Materials and Methods

The study was carried out on inhabitants aged 71–80 years in south-eastern Poland (Podkarpackie region). The personal data (name, address, and ID number) of 25,000 individuals was selected randomly from the database of the Ministry of the Interior and Administration in Warsaw. From this group, the data was allocated randomly into a main sample (800 people), and, as a result of an additional draw, an additional random sample (12200 people) was taken from the reserve sample (24200 people). Simple random sampling was performed, without returns of the selected respondents, with the help of SPSS software. The study was representative, with an assumed confidence level of 95% (0.95) and an estimated error (maximum error) of 3% [26].

**Figure 1:**
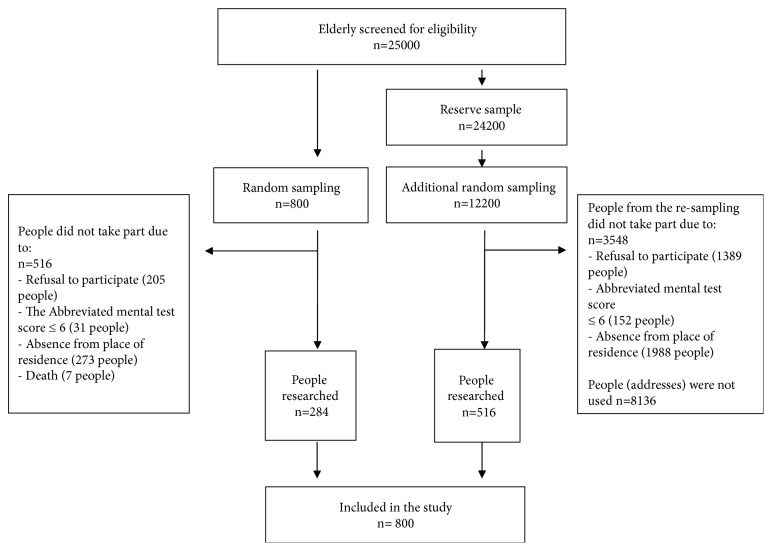
Flow diagram of the study in people aged 71–80 years in south-eastern Poland.

Figure 1 shows a flow diagram of the study selection process.

### 2.1. Procedures

The study took place at the turn of 2015 and 2016. It was surveyed by means of direct interviews carried out by qualified and trained interviewers at the residences of the respondents. The participants met the following criteria: age 71–80 years, normal cognitive status measured by AMTS (abbreviated mental test score >6), and informed consent.

### 2.2. Ethics

In accordance with the Declaration of Helsinki, the participants were informed about the aim and the course of the study and gave their informed consent to take part. The study design was approved by the Bioethical Committee of the University of Rzeszow (Resolution No. 18/03/2015).

### 2.3. Data Collection

Data was collected using the 36*-*item interviewed-administered WHODAS 2.0 questionnaire, based on the WHO Disability Assessment Schedule. Additionally, a second questionnaire was used to collect information concerning age, sex, height, body mass, place of residence, marital status, education, income, physical activity, social activity, functioning in the environment, and health.

The WHODAS 2.0 questionnaire is used to measure functioning, disability and health. Its aim is to determine the level of functioning in six domains: cognition, mobility, self-care, getting along, life activities—domestic responsibilities—and participation in community activities [19]. The life activity, work and school domain, was not included in the study as it does not concern the studied population.

WHODAS 2.0 contained 36 items. Responses were given on a scale from 1 to 5, where 1 is no; 2 is mild; 3 is moderate; 4 is severe; and 5 is extreme difficulties. According to the instruction manual [19], the multisectional positions were coded and the original score was converted to a scale ranging from 0 to 100, in which higher scores indicated more limitations (0 = no difficulty; 100 = very high degree of difficulty). Finally, in order to determine the overall level of disability and disability in specific domains of WHODAS 2.0, the following ICF-compatible scale was used: no disability (0–4%), mild disability (5–24%), moderate disability (25–49%), severe disability (50–95%), and extreme disability (96–100%) [10]. The questionnaire had previously been qualified in groups of seniors in Poland, achieving very high psychometric scores [27].

### 2.4. Statistical Analysis

Statistical analysis was performed using Statistica software version 13.1. Descriptive statistics were calculated from the data. The Shapiro-Wilk test was then used to determine the normal distribution of the quantitative data. Following this, the chi-squared test (for qualitative variables) and the Mann–Whitney *U* test (for quantitative data) were used for preliminary analysis of the relationships between the set of tested variables and at least moderate disability. A logistic regression model was then used to identify the factors related to at least moderate disability identified by WHODAS 2.0. Due to its significant impact on the functioning of older people in everyday life, the marginal value of the disability level was chosen as at least moderate [10]. The model was a good fit to the data, as demonstrated by the results of the Hosmer-Lemeshow test (*χ*^2^_*HL*_ = 13.66, *p* = 0.09) and the pseudo *R*^2^ value of 0.7725, indicating that the model correctly classified 77.25% of cases. All parameters of the model were found to be statistically significant. Statistical significance was set at *p* < 0.05.

## 3. Results

The characteristics of the studied population are presented in Table 1.

The study included 800 older adults (470 females and 330 males) aged 71–80 years, mean age 75.4 years (SD = 2.9 years): 75.5 years in women (SD = 2.9 years) and 75.4 years in men (SD = 3.0 years). Almost half of the respondents (46.75%) lived in cities and the remainder (53.25%) in the countryside. Most respondents had a primary education (43.50%), although others reported a vocational (20.75%), secondary vocational (18.75%), secondary comprehensive (7.75%), or tertiary (9.75%) education. Only 7.75% of the respondents reported no disability or functional limitations. Of the remainder, moderate level disabilities were most commonly observed (43.88%), followed by those of mild (30.62%) and severe level (17.25%). Extremely high levels of disability were found in only 0.5% of the respondents.

The mean level of disability measured by WHODAS 2.0 was 32.90 (SD = 20.81). The highest mean level of disability was found in the life domain (mean = 41.63, SD = 28.23), with severe (38.75%) and extremely high (5.62%) disabilities also being reported in this area. The next highest level of disability was found in participation in social life (mean = 36.61, SD = 21.84), followed by getting along (mean = 35.80, SD = 27.50) and problems associated with mobility (mean = 35.25; SD = 27.40). Problems with taking care of oneself, personal hygiene, getting dressed, eating, and staying alone at home (mean = 16.84; SD = 22.53) caused the least problems (Table 1).

Our preliminary analysis identified 10 sociodemographic variables that were significantly related to the dependent variable, defined as the occurrence of at least a moderate level of disability (at *p* < 0.05). Disability was found to be unrelated to sex, BMI, and performing everyday physical activity for at least 150 minutes per week (Table 2).

A detailed analysis using the logistic regression model revealed the existence of a relationship between the occurrence of at least a moderate level of disability and age, the number of chronic diseases, the level of education, and performing physical exercises (Table 3). Nonadaptation of the place of residence of the respondents was the factor most strongly related to at least a moderate disability level, increasing its chances of occurring by more than sevenfold (OR = 7.81); other factors included no opportunity to use help (OR = 1.56), a vocational level of education (OR = 1.40), age (OR = 1.17), and number of chronic diseases (OR = 1.14) and do not perform physical exercises (OR = 0.38; OR = 0.18) (Table 3).

## 4. Discussion

The studied group, comprising older people aged 71–80 years in south-eastern Poland, demonstrated a very high incidence of disability. Only 7.75% of the respondents reported no disability or any limitations in functioning, whereas 30.42% had a mild level of disability. In addition, 61.63% of the subjects demonstrated at least a moderate level of disability, of whom 17.75% displayed severe or extreme disability. The mean level of disability was found to be 32.90 according to WHODAS 2.0 (SD = 20.81). A similarly high level of disability was found in the same age group in India, with a mean score of 34.2 (SD = 20.6) for those aged 70–75 years and a mean score of 39.8 (SD = 18.6) for those aged 75–80 years. Similar levels of disability were also found in the Russian Federation (with a mean score of 27.0 [SD = 16.4] for those aged 70–75 years, and 34.0 [SD = 20.0] for 75–80 years) and in Ghana (mean score of 28.2 [SD = 18.4] for those 70–75 years and 33.0 [SD = 19.8] for those 75–80 years). However, a significantly lower level of disability was found in China (mean score of 12.4 [SD = 13.4] for those aged 70–75 years; 18.0 [SD = 16.1] for those 75–80 years) and in Mexico (mean score of 18.9 [SD = 13.4] for those 70–75 years; 19.9 [SD = 18.4] for those 75–80 years) [18].

The highest mean level of disability was found in life activities (mean score = 41.63, SD = 28.23), with the highest scores for severe and extremely high disability being 38.75 and 5.62, respectively. Lower effectiveness or speed may endanger the safety and independence of older people when performing tasks [28]. The observed decrease in functioning may also be associated with a lack of adaptation of the home to reduced functional abilities and the lack of any possibility for getting help.

The relationship between the mean level of disability and participation in social life was 36.61 (SD = 21.84). Functional ability, social relationships, and participation all decline with age. Indeed, limited functioning may affect the ability to maintain social links [29]. Social engagement, life activities, and maintaining social relationships improve self-esteem and identity and may have a beneficial effect on health of older people [30]. Our findings suggest that older people in a larger and denser social network live a healthier lifestyle [31].

In the investigated group, a high level of disability in maintaining relationships and communication with others (mean = 35.80, SD = 27.50) was found. Physical limitations, rapid changes in family structure, and communication difficulties between generations may lead to older people facing social isolation [32]. Social engagement gives a sense of purpose and control over one's life. Pavey et al. [33] report that older people who were isolated from society and who report watching television for many hours a day run a higher risk of death than those who remain socially active. Kono et al. [34] indicate that a reduced frequency of going out was an important predictor of an increased risk of deterioration in health in people with functional and psychosocial problems: it seems that going out more frequently may have beneficial therapeutic effects for older people.

Another analysed issue was the relationship between disability and mobility (mean = 35.25, SD = 27.40). Walking speed is an important predictor for disability in daily activities, necessity of medical care, hospitalization, placement in a nursing home, and mortality [35]. A decrease in walking speed regardless of baseline speed is an important predicator for disability and dependence [36]. Improving or maintaining a proper level of mobility, both at home and outside, is closely linked to the lowering of barriers in the surrounding environment [37].

To assess the odds of disability in the investigated group, the following factors were analysed: age, number of chronic disease, education, performing physical exercises to strengthen muscles and and improve fitness, the possibility of getting help from others in everyday life, and the adaptation of a home to the needs of everyday functioning. We found that, with increasing age, the odds of at least moderate disability increased. Similar results were obtained by Connolly et al., who found that, after 65 years of age, the chance of disability increased twice every five years [38]. Our present findings also show that, with any subsequent illness, the chance of at least a moderate level of disability increased. Similarly, Fried et al. pointed out that the level of disability among the elderly increases with the number of chronic diseases [39].

Persons who could get help from others also had over an increased chance of becoming disabled than those who did not (OR = 1.56; 95% CI = 1.04–2.33). In addition, those with at most a vocational education had a higher odds of developing at least a moderate level of disability compared to those with at least a secondary education (OR = 1.40; 95% CI = 1.01–2.08). A similar relationship between education and disability has been observed in China, Russia [18], and Singapore [40].

In the surveyed population, the odds of a moderate level of disability were more than seven times higher in respondents who declared that their place of residence was not fully adapted to their functional needs than in those living in a modified apartment, e.g., with worktops and cupboards placed at an appropriate height, the thresholds removed, and handholds and good lighting installed. Similar results were obtained by Parmelee et al. [41], who showed that older people living in nonadapted homes are at a greater risk of developing disability. Stark [42] reports that removing architectural barriers from the homes of older people may significantly improve their functioning and ability to perform daily activities. In 1996, Morini et al. [43] observed a tendency towards increased market demand for housing adaptation to meet the needs of older adults in Italy, France, the United Kingdom, and the Netherlands. In 2010, a report published by the Expert Group of the European Commission [44] on Full Accessibility pointed out the need to increase the accessibility of buildings and sites designed and managed as safe, healthy, comfortable, and adapted for the use of older people.

In the studied population, those aged 71–80 years who did not exercise had a higher chance of becoming moderately disabled than those who exercised at least four times a week. Maintaining high physical fitness is a key factor in successful aging [45]. Dunlop et al. [46] showed that people sitting for nine hours during the day runs a 46% greater risk of developing a disability in activities of daily living and found a strong relationship between disability and physical activity in an American population. A meta-analysis by Tak et al. [7] also showed that physical activity slows the aging process and reduces the incidence of disability and dependence. In a systematic review, Vermeulen and Neyers [47] formulated a disability forecast for older adults and factors that significantly contribute to functional impairment; this included lower walking speed, reduced grip strength, and physical activity, as well as weight loss.

### 4.1. Limitations

There were some limitations to our study. It was a cross-sectional study, so we were not able to show the cause and effect relationship between disability and variables that could affect our results. Additional research with longitudinal data would enable better understanding of developing disability, and the role of complex set of variables in this process. The fact that the investigations were carried out in a single selected region, i.e., south-eastern Poland, is a limitation of the study. However, the obtained results may be extrapolated to the whole 71- to 80-year-old population of Poland, as the population in south-eastern Poland is generally typical of those of the country as a whole.

## 5. Conclusion

The obtained results could serve as a basis for designing a genuine strategy for health policy for older people in Poland. Improving medical care, health education, adjusting the living environment of older people to their current functional status, such as their housing conditions, and providing greater access to help from others, together with greater promotion of physical activity, could reduce the disability and dependency of people aged 71–80 years. The care of older adults should be based on a team of specialists focused on personalized and comprehensive medical care. As older people are treated by primary health care, the GP should also be responsible for the health education of older patients with lower levels of education, as noted by Barnett et al. [48]. The introduction of programs intended to activate older adults should be preceded by the identification of priority areas that require most support. Our findings indicate the need to intensify the activities aimed at improving the performance of complex daily activities such as cleaning, cooking, and vacuuming to enable older people to carry out their own household duties. Other areas that require the most attention include encouraging participation in social life and relationships with the environment. It is necessary to involve older people in group activities such as interest clubs or volunteering, which will strengthen social relationships and help maintain the interpersonal harmony of the elderly. Moreover, it is worth considering a systemic solution which may guarantee free physical activities run by physiotherapists, e.g., in health centers. The cost of prevention is much lower than the cost of treatment or care of disabled and dependent older people.

## Figures and Tables

**Table 1:** Characteristics of the studied population.

		Mean (SD) N (%)
***Sociodemographic features***		
Age (years)		75.4 (2.9)
Body Mass Index (BMI)		27.21 (4.65)
Number of chronic diseases		5.54 (3.36)
Sex	Female	470 (58.75)
	Male	330 (41.25)
Education	Primary	348 (43.50)
	Vocational	166 (20.75)
	Secondary comprehensive	62 (7.75)
	Secondary vocational	146 (18.25)
	Tertiary	78 (9.75)
Place of residence	City/town	374 (46.75)
	Countryside	426 (53.25)

***Disability***		
Disability domains WHODAS 2.0	Cognitive functions	28.87 (22.04)
	Mobility	35.25 (27.40)
	Self-care	16.84 (22.53)
	Getting along	35.80 (27.05)
	Life activities	41.63 (28.23)
	Participation in community life	36.61 (21.84)
	Total WHODAS 2.0	32.90 (20.81)
Overall level of disability	None	62 (7.75)
	Mild	245 (30.62)
	Moderate	351 (43.88)
	Severe	138 (17.25)
	Extreme high	4 (0.50)

***Physical activity of the respondents***		
Physical activity performed daily a minimum of 150 minutes per week	No	553 (69.12)
	Yes	247 (30.88)
		
Physical exercises performed to strengthen muscles and improve physical performance	None	627 (78.38)
	One to three times per week	73 (9.12)
	Four and more times per week	100 (12.50)

***Social activity of the respondents***		
Membership in at least one organization/group/association	No	673 (84.12)
	Yes	127 (15.88)
		
Work after retirement	No	712 (89.00)
	Yes	88 (11.00)

***Functioning of the respondents in the environment***		
Opportunity to use the help of other people in everyday life	No	219 (27.38)
	Yes	581 (72.62)
Adaptation of the interior of a flat/house to the needs of everyday functioning	Not fully adapted	598 (74.75)
	Fully adapted	202 (25.25)
Adaptation of the residential environment to the needs of everyday functioning	Not fully adapted	604 (75.50)
	Fully adapted	196 (24.50)

**Table 2:** Analysis of factors related to the level of disability according to the results of the WHODAS 2.0 questionnaire.

		**Disabled with a minimum moderate level of disability** **Mean (SD)** **N (%)**	**Able or disabled with a mild disability** **Mean (SD)** **N (%)**	**p value**
***Sociodemographic features***				
Age		74.61 (2.72)	76.00 (2.90)	**0.001** ^a^
BMI		26.76 (3.08)	27.50 (5.01)	0.086^a^
Number of chronic diseases		5.07 (3.30)	5.83 (3.36)	**<0.002** ^a^
Sex	Female	301 (64.04)	169 (35.96)	0.093^b^
	Male	192 (58.18)	138 (41.82)	
Education	At most vocational	350 (68.09)	164 (31.91)	**<0.001** ^b^
	At least secondary	143 (50.00)	143 (50.00)	
Place of residence	City	216 (57.75)	158 (42.25)	**0.034** ^b^
	Countryside	277 (65.02)	143 (34.98)	
***Physical activity of the respondents***				
Performing everyday physical activity for at least 150 minutes a week	Yes	141 (57.09)	106 (42.91)	0.077^b^
	No	352 (63.65)	201 (36.35)	
Performing physical exercises to strengthen muscles and improve physical performance	One to three times a week	438 (69.86)	189 (30.14)	**<0.001** ^b^
	Four or more times a week	34 (46.58)	39 (53.42)	
	No	21 (21.00)	79 (79.00)	
***Social activity of the respondents***				
Membership in at least one organization/group/association	Yes	429 (63.74)	244 (36.26)	**0.004** ^b^
	No	64 (50.39)	63 (49.61)	
Work after retirement	Yes	43 (48.86)	45 (51.14)	**0.009** ^b^
	No	450 (63.20)	262 (36.80)	
***Functioning of the respondents in the environment***				
Opportunity to use help of other people in everyday life	Yes	331 (56.97)	250 (43.03)	**0.001** ^b^
	No	162 (73.97)	57 (26.03)	
Adaptation of the flat/house to the needs of everyday functioning	Fully adapted	51 (25.25)	151 (74.75)	**<0.001** ^b^
	Not fully adapted)	442 (73.91)	156 (26.09)	
Adaptation of the residential environment to the needs of everyday functioning	Fully adapted	48	148	**0.001** ^b^
	Not fully adapted	445	159	

^a^Mann-Whitney *U* Test.

^b^Chi-squared Test.

**Table 3:** Logistic regression model showing factors related to at least a moderate disability level.

		**Disabled with a minimum moderate level of disability**		
***Variable***		**Odds ratio**	**95% CI**	**p value**
Age		1.17	1.10–1.25	**<0.001**
Number of chronic diseases		1.14	1.08–1.21	**<0.001**
Education	At least vocational level (versus at least secondary comprehensive level)	1.40	1.01–2.08	**0.040**
Performing exercises	One to three times a week (versus no exercise)	0.38	0.21–0.69	**0.001**
	Four and more times a week (versus no exercise)	0.18	0.10–0.32	**<0.001**
Home adaptation	Not fully adapted (versus fully adopted)	7.81	5.07–12.00	**<0.001**
Opportunity to use help of other people	No (versus those with the opportunity to use help of other people)	1.56	1.04–2.33	**0.030**

## References

[1] Heitger M. H., Goble D. J., Dhollander T. (2013). Bimanual Motor Coordination in Older Adults Is Associated with Increased Functional Brain Connectivity - A Graph-Theoretical Analysis. *PLoS ONE*.

[2] ec.europa.eu Population Structure and Ageing.

[3] Żołędowski C. (2013). *Aging population - Poland against the background of the European Union*.

[4] The Central Statistical Office (2015). *Population forecast for 2014-2015. Studies and statistical analysis*.

[5] Beck O., Łakomski M., Kędziora–Kornatowska K. Health determinants of disability of the elderly.

[6] The Central Statistical Office (2014). *Health and health behavior of the inhabitants of Poland in the light of the EHIS study*.

[7] Tak E., Kuiper R., Chorus A., Hopman-Rock M. (2013). Prevention of onset and progression of basic ADL disability by physical activity in community dwelling older adults: a meta-analysis. *Ageing Research Reviews*.

[8] Chatterji S., Byles J., Cutler D., Seeman T., Verdes E. (2015). Health, functioning, and disability in older adults—present status and future implications. *The Lancet*.

[9] Mossakowska M., Wiecek A., Bledowski P. (2012). *Medical, psychological and socioeconomic aspects of aging in Poland*.

[10] Salomon J. A., Wang H., Freeman M. K. (2012). Healthy life expectancy for 187 countries, 1990–2010: a systematic analysis for the Global Burden Disease Study 2010. *The Lancet*.

[11] World Health Organization (2001). *International Classification of Functioning, Disability and Health*.

[12] McDaid D., Cieza A., Gomez A. R. (2009). Bridging knowledge: reflections on crossing the boundaries between long-term care and support. *International Journal of Integrated Care*.

[13] Federici S., Meloni F., Mancini A., Lauriola M., Olivetti Belardinelli M. (2009). World Health Organisation Disability Assessment Schedule II: Contribution to the Italian validation. *Disability and Rehabilitation*.

[14] Chang K.-H., Liao H.-F., Yen C.-F. (2015). Association between muscle power impairment and WHODAS 2.0 in older adults with physical disability in Taiwan. *Disability and Rehabilitation*.

[15] Almazán-Isla J., Comín-Comín M., Damián J. (2014). Analysis of disability using WHODAS 2.0 among the middle-aged and elderly in Cinco Villas, Spain. *Disability and Health Journal*.

[16] Donmez L., Gokkoca Z., Dedeoglu N. (2005). Disability and its effects on quality of life among older people living in Antalya city center, Turkey. *Archives of Gerontology and Geriatrics*.

[17] Adib-Hajbaghery M., Aghahoseini S. (2007). The evaluation of disability and its related factors among the elderly population in Kashan, Iran. *BMC Public Health*.

[18] Biritwum R. B., Minicuci N., Yawson A. E. (2016). Prevalence of and factors associated with frailty and disability in older adults from China, Ghana, India, Mexico, Russia and South Africa. *Maturitas*.

[19] Üstün TB., Kostanjsek N., Chatterji S. (2010). *Measuring Health and Disability Manual for WHO Disability Assessment Schedule WHODAS 2.0*.

[20] Federici S., Bracalenti M., Meloni F., Luciano J. V. (2017). World Health Organization disability assessment schedule 2.0: An international systematic review. *Disability and Rehabilitation*.

[21] Olaya B., Moneta M. V., Koyanagi A. (2016). The joint association of depression and cognitive function with severe disability among community-dwelling older adults in Finland, Poland and Spain. *Experimental Gerontology*.

[22] Ćwirlej-Sozańska A., Wilmowska-Pietruszyńska A., Wiśniowska-Szurlej A., Sozański B. (2017). Analysis of health, functioning and disability of rural inhabitants aged 60-80 living in south-eastern Poland – a cross sectional study. *Annals of Agricultural and Environmental Medicine*.

[23] Perales J., Martin S., Ayuso-Mateos J. L. (2014). Factors associated with active aging in Finland, Poland, and Spain. *International Psychogeriatrics*.

[24] Błędowski P., Mossakowska M., Wiecek A., Błędowski P. (2035). Aging as a social problem. Perspectives of demographic aging of the Polish population by 2035. *Medical, psychological and socioeconomic aspects of aging in Poland*.

[25] Lin S.-F., Beck A. N., Finch B. K., Hummer R. A., Master R. K. (2012). Trends in US older adult disability: Exploring age, period, and cohort effects. *American Journal of Public Health*.

[26] Sawiński Z., Fieldwork J. S., Sztabiński P. S., Sawiński Z., Sztabiński F. (2005). Metody doboru respondentów. *Wydawnictwo Instytut Filozofii i Socjologii Polskiej*.

[27] Ćwirlej-Sozańska A., Wilmowska-Pietruszyńska A., Sozański B. (2017). Validation of the Polish version of the World Health Organization Disability Assessment Schedule (WHODAS 2.0) in an elderly population (60–70 years old). *International Journal of Occupational Safety and Ergonomics*.

[28] Guralnik J. M., Ferrucci L., Simonsick E. M., Salive M. E., Wallace R. B. (1995). Lower-extremity function in persons over the age of 70 years as a predictor of subsequent disability. *The New England Journal of Medicine*.

[29] Avlund K., Due P., Holstein B. E. (2002). Changes in social relations in old age. Are they influenced by functional ability?. *Aging Clinical and Experimental Research*.

[30] Berkman L. F., Glass T., Brissette I., Seeman T. E. (2000). From social integration to health: Durkheim in the new millennium. *Social Science & Medicine*.

[31] Bot S. D., Mackenbach J. D., Nijpels G., Lakerveld J. (2016). Association between social network characteristics and lifestyle behaviours in adults at risk of diabetes and cardiovascular disease. *PLoS ONE*.

[32] Kashikar Y., Nagarkar A. (2016). Prevalence and Determinants of Frailty in Older Adults in India. *Indian Journal of Gerontology*.

[33] Pavey T. G., Peeters G. M. E. E., Brown W. J. (2015). Sitting-time and 9-year all-cause mortality in older women. *British Journal of Sports Medicine*.

[34] Kono A., Kai I., Sakato C., Rubenstein L. Z. (2004). Frequency of Going Outdoors: A Predictor of Functional and Psychosocial Change among Ambulatory Frail Elders Living at Home. *The Journals of Gerontology. Series A, Biological Sciences and Medical Sciences*.

[35] Albert S. M., Bear-Lehman J., Anderson S. J. (2015). Declines in mobility and changes in performance in the instrumental activities of daily living among mildly disabled community-dwelling older adults. *The Journals of Gerontology. Series A, Biological Sciences and Medical Sciences*.

[36] Artaud F., Singh-Manoux A., Dugravot A., Tzourio C., Elbaz A. (2015). Decline in fast gait speed as a predictor of disability in older adults. *Journal of the American Geriatrics Society*.

[37] Levasseur M., Généreux M., Bruneau J.-F. (2015). Importance of proximity to resources, social support, transportation and neighborhood security for mobility and social participation in older adults: Results from A Scoping Study. *BMC Public Health*.

[38] Connolly D., Garvey J., McKee G. (2017). Factors associated with ADL/IADL disability in community dwelling older adults in the Irish longitudinal study on ageing (TILDA). *Disability and Rehabilitation*.

[39] Hung W. W., Ross J. S., Boockvar K. S., Siu A. L. (2012). Association of chronic diseases and impairments with disability in older adults: A decade of change?. *Medical Care*.

[40] Mahesh M., Abdin E., Vaingankar J. A. (2016). Disability in Singapore’s elderly population. *Annals, Academy of Medicine, Singapore*.

[41] Parmelee P. A., Lawton M. P., Birren J. E., Schaie K. W. (1994). The design of special environments for the aged. *Handbook of the psychology of ageing*.

[42] Stark S. (2004). Removing Environmental Barriers in the Homes of Older Adults with Disabilities Improves Occupational Performance. *OTJR: Occupation, Participation and Health*.

[43] Morini A., Pomposini R., Ural O., Altýnbilek D., Birgönül T. New designs for aged people housing.

[44] accessibletourism.org, A Europe accessible for all, 2010

[45] Lin P.-S., Hsieh C.-C., Cheng H.-S., Tseng T.-J., Su S.-C. (2016). Association between physical fitness and successful aging in taiwanese older adults. *PLoS ONE*.

[46] Dunlop D. D., Song J., Arntson E. K. (2015). Sedentary time in US older adults associated with disability in activities of daily living independent of physical activity. *Journal of Physical Activity & Health*.

[47] Vermeulen J., Neyens J. C., van Rossum E., Spreeuwenberg M. D., de Witte L. P. (2011). Predicting ADL disability in community-dwelling elderly people using physical frailty indicators: a systematic review. *BMC Geriatrics*.

[48] Barnett K., Mercer S. W., Norbury M., Watt G., Wyke S., Guthrie B. (2012). Epidemiology of multimorbidity and implications for health care, research, and medical education: a cross-sectional study. *The Lancet*.

